# Supplementary material for: Health Professionals’ Knowledge and Views on the Use of Convenience Cooking Products: An Australian Cross-Sectional Study
Source: Nutrients. 2025 Mar 26;17(7):1156. doi: 10.3390/nu17071156 (PMC11990915; doi:10.3390/nu17071156)
Supplement: Supplementary file 1 [file nutrients-17-01156-s001.zip › nutrients-3541407-supplementary.pdf]

STROBE Statement—checklist of items that should be included in reports of observational studies.

	Item No.	Recommendation	Page No.	Relevant text from manuscript
**Title and abstract**	1	(*a*) Indicate the study's design with a commonly used term in the title or the abstract	1	Study design is indicated in the methods/findings section of the abstract: "A cross-sectional survey analysed 143 Australian health professional participants, including dietitians, nutritionists, and doctors."
		(*b*) Provide in the abstract an informative and balanced summary of what was done and what was found	1	The following information is stated in the study abstract: study objective, methods, and results.
**Introduction**				
Background/rationale	2	Explain the scientific background and rationale for the investigation being reported	1-3	The rationale and the existing literature are presented.
Objectives	3	State specific objectives, including any prespecified hypotheses	3	A statement at the end of the introduction specifies primary and secondary objectives. Primary objectives: to explore the opinions Australian health professionals have regarding convenience cooking products and if they recommend these products to their clients or if they would recommend them to their clients. Secondary objectives: to analyse their knowledge of decision

				fatigue and if these products could provide an alternative when faced with decision fatigue.
**Methods**				
Study design	4	Present key elements of study design early in the paper	4	The study design is stated under heading 2.2: Study Design, Setting, and Recruitment. The key elements describe why the following method was used.
Setting	5	Describe the setting, locations, and relevant dates, including periods of recruitment, exposure, follow-up, and data collection	4	Setting, location, relevant dates (period of recruitment), exposure, and data collection are all described in the methods under Section *2.2: Study Design, Setting, and Recruitment*. No follow-up was required.
Participants	6	(*a*) *Cohort study*—Give the eligibility criteria, and the sources and methods of selection of participants. Describe methods of follow-up<br>*Case-control study*—Give the eligibility criteria, and the sources and methods of case ascertainment and control selection. Give the rationale for the choice of cases and controls<br>*Cross-sectional study*—Give the eligibility criteria, and the sources and methods of selection of participants	4	The study population is described in the methods section. "Participants were included in the study if they were living in Australia, over 18 years of age, and were professionals working in dietetics, nutrition, medicine, nursing, or any other allied health field where they are involved in nutrition."

		(*b*) *Cohort study*—For matched studies, give matching criteria and number of exposed and unexposed<br>*Case-control study*—For matched studies, give matching criteria and the number of controls per case		
Variables	7	Clearly define all outcomes, exposures, predictors, potential confounders, and effect modifiers. Give diagnostic criteria, if applicable	4	All variables are described in the methods.
Data sources/ measurement	8*	For each variable of interest, give sources of data and details of methods of assessment (measurement). Describe comparability of assessment methods if there is more than one group	3-4	Data collection and measurement were the same for all variables and are described in the methods section. Data were collected via an online cross-sectional survey (QuestionPro). Participants self-reported. Responses were measured using categorical variables and Likert scales (1 = strongly disagree to 5 = strongly agree). To ensure comparability, all participants completed the same standardized questionnaire, with reverse-scored Likert items as attention checks.
Bias	9	Describe any efforts to address potential sources of bias	3	Potential bias is listed in 2.2: Study Design, Setting, and Recruitment.
Study size	10	Explain how the study size was arrived at	3-4	The methods explain the study size. The final sample included 143 participants, after excluding ineligible responses. A formal sample size calculation was not

				performed due to the exploratory nature of the study.
Quantitative variables	11	Explain how quantitative variables were handled in the analyses. If applicable, describe which groupings were chosen and why	3-4	Quantitative variables (e.g., age, years of experience, frequency of product use) were categorized for analysis.
Statistical methods	12	(*a*) Describe all statistical methods, including those used to control for confounding	5	These are described in the method section. Both categorical and continuous data were used. The statistically significant threshold was a p-value of <0.05. Contingency tables (Pearson X2) and nominal logistical regression were used to assess the differences in distributions between categories. Standard least squares regression was used to compare adjusted least squares means by category (adjusted for age, sex, income, education, and working hours).
		(*b*) Describe any methods used to examine subgroups and interactions	5	These are described in the method section. Contingency tables (Pearson X2) and nominal logistical regression were used to assess the differences in distributions between categories. Standard least squares regression was used to compare adjusted least squares means by category (adjusted for age, sex,

				income, education, and working hours).
		(*c*) Explain how missing data were addressed	4	This was described in the methods section: "Any incomplete or missing values were automatically considered invalid and were not included in the study."
		(*d*) *Cohort study*—If applicable, explain how loss to follow-up was addressed<br>*Case-control study*—If applicable, explain how matching of cases and controls was addressed<br>*Cross-sectional study*—If applicable, describe analytical methods taking account of sampling strategy	N/A	Not applicable.
		(*e*) Describe any sensitivity analyses	N/A	Not applicable.
**Results**				
Participants	13*	(a) Report numbers of individuals at each stage of study—eg numbers potentially eligible, examined for eligibility, confirmed eligible, included in the study, completing follow-up, and analysed	5	This is described at the beginning of the results section: "A total of 272 participants responded to the survey. One hundred and thirty-eight participants were excluded due to incomplete responses, twelve were excluded for completing the survey in less than half the median completion time (<450 s), and a further eight were excluded for not residing in Australia. Overall, there were a total of 143 participants in the final sample".
		(b) Give reasons for non-participation at each stage	5	This is described at the beginning of the results section: "A total of 272 participants responded to the survey. One hundred and thirty-eight participants were excluded

				due to incomplete responses, twelve were excluded for completing the survey in less than half the median completion time (<450 s), and a further eight were excluded for not residing in Australia. Overall, there were a total of 143 participants in the final sample".
		(c) Consider use of a flow diagram	N/A	Not applicable.
Descriptive data	14*	(a) Give characteristics of study participants (eg demographic, clinical, social) and information on exposures and potential confounders	5-6	Results 3.1 Demographics and Table 1 describe the participants and their profession.
		(b) Indicate number of participants with missing data for each variable of interest	6	The total recorded data for each variable are stated in the variable headline of the table.
Outcome data	15*	*Cohort study*—Report numbers of outcome events or summary measures over time		
		Case-control study—Report numbers in each exposure category, or summary measures of exposure		
		Cross-sectional study—Report numbers of outcome events or summary measures	*6*	All numbers are reported in the Tables.
Main results	16	(*a*) Give unadjusted estimates and, if applicable, confounder-adjusted estimates and their precision (eg, 95% confidence interval). Make clear which confounders were adjusted for and why they were included	6-8	The study presents both unadjusted and adjusted estimates. These are described in the results.<br>To account for potential confounding factors, the study conducted an adjusted analysis using standard least squares regression.
		(*b*) Report category boundaries when continuous variables were categorized	5-7	Category boundaries are displayed in variable headings in tables where applicable (age, years of

			experience, and frequency of recommendation).
	(*c*) If relevant, consider translating estimates of relative risk into absolute risk for a meaningful time period	N/A	Not applicable.

Continued on next page

Other analyses	17	Report other analyses done—eg analyses of subgroups and interactions, and sensitivity analyses	N/A	Not applicable.
**Discussion**				
Key results	18	Summarise key results with reference to study objectives	8 & 10	Key results are described at the beginning of the discussion section, as well as being summarised in the conclusion.
Limitations	19	Discuss limitations of the study, taking into account sources of potential bias or imprecision. Discuss both direction and magnitude of any potential bias	11	THe limitations are described under the limitations and future directions sub-heading in the conclusion.
Interpretation	20	Give a cautious overall interpretation of results considering objectives, limitations, multiplicity of analyses, results from similar studies, and other relevant evidence	8-11	These aspects were discussed or referenced where possible and limitations were taken into consideration.
Generalisability	21	Discuss the generalisability (external validity) of the study results	11	This is discussed in the conclusion under the sub-heading Limitations and Future Directions.
**Other information**				
Funding	22	Give the source of funding and the role of the funders for the present study and, if applicable, for the original study on which the present article is based	11	This research received no external funding.
